# Supplementary material for: The complete chloroplast genome sequence of strawberry (Fragaria × ananassa Duch.) and comparison with related species of Rosaceae
Source: PeerJ. 2017 Oct 12;5:e3919. doi: 10.7717/peerj.3919 (PMC5641433; doi:10.7717/peerj.3919)
Supplement: File S1 [file peerj-05-3919-s001.docx]

| **Primer** | **Forward sequence (5´to 3´)** | **Reverse sequence (5´to 3´)** |
| --- | --- | --- |
| LSC/IRB | AGTTATGAACCCTGTAGACC | CAAGGCAGTGGATTGTGA |
| LSC/IRA | AATGCTCCTTTCCGTTAT | AACCCTGTAGACCATCCC |
| SSC/IRB | TTCCATCGTTTAGGGTCG | GGTTGGGTTGGTATTAGG |
| SSC/IRA | GGGACTCAAGAACAAGAA | TAATCGCGGTTACATAGA |
