# Supplementary material for: The complete chloroplast genome sequence of strawberry (Fragaria × ananassa Duch.) and comparison with related species of Rosaceae
Source: PeerJ. 2017 Oct 12;5:e3919. doi: 10.7717/peerj.3919 (PMC5641433; doi:10.7717/peerj.3919)
Supplement: File S2 [file peerj-05-3919-s002.docx]

| **Gene** | **Location** | **Exon I (bp)** | **Intron I (bp)** | **Exon II (bp)** | **Intron II (bp)** | **Exon III (bp)** |
| --- | --- | --- | --- | --- | --- | --- |
| *clpP* | LSC | 69 | 827 | 294 | 662 | 228 |
| *ndhA* | SSC | 543 | 1191 | 540 |  |  |
| *ndhB* | IR | 777 | 678 | 756 |  |  |
| *petB* | LSC | 6 | 772 | 648 |  |  |
| *petD* | LSC | 9 | 716 | 474 |  |  |
| *rps12** | LSC | 114 | - | 232 | 538 | 26 |
| *rpl2* | IR | 390 | 683 | 435 |  |  |
| *rpl16* | LSC | 9 | 993 | 402 |  |  |
| *rpoC1* | LSC | 432 | 758 | 1611 |  |  |
| *rps16* | LSC | 42 | 858 | 228 |  |  |
| *trnA*-*UGC* | IR | 38 | 813 | 35 |  |  |
| *trnG*-*GCC* | LSC | 23 | 694 | 48 |  |  |
| *trnK*-*UUU* | LSC | 37 | 2497 | 35 |  |  |
| *trnL*-*UAA* | LSC | 35 | 422 | 50 |  |  |
| *trnI*-*GAU* | IR | 42 | 954 | 35 |  |  |
| *trnV*-*UAC* | LSC | 39 | 600 | 35 |  |  |
| *ycf3* | LSC | 126 | 723 | 228 | 807 | 153 |

**Note.**

***The *rps12* is a trans-spliced gene with the 5’ end located in the LSC region and duplicated in the 3’ end in the IR regions.
