# Supplementary material for: The complete chloroplast genome sequence of strawberry (Fragaria × ananassa Duch.) and comparison with related species of Rosaceae
Source: PeerJ. 2017 Oct 12;5:e3919. doi: 10.7717/peerj.3919 (PMC5641433; doi:10.7717/peerj.3919)
Supplement: File S3 [file peerj-05-3919-s003.docx]

| **Amino acid** | **Codon** | **No.** | **RSCU** | **tRNA** | **Amino acid** | **Codon** | **No.** | **RSCU** | **tRNA** |
| --- | --- | --- | --- | --- | --- | --- | --- | --- | --- |
| Phe | UUU | 883 | 1.36 |  | Tyr | UAU | 682 | 1.61 |  |
| Phe | UUC | 414 | 0.64 | *trnF*-*GAA* | Tyr | UAC | 166 | 0.39 | *trnY*-*GUA* |
| Leu | UUA | 819 | 2.04 | *trnL*-*UAA* | Stop | UAA | 0 | 0 |  |
| Leu | UUG | 474 | 1.18 | *trnL*-*CAA* | Stop | UAG | 0 | 0 |  |
| Leu | CUU | 503 | 1.25 |  | His | CAU | 401 | 1.48 |  |
| Leu | CUC | 148 | 0.37 |  | His | CAC | 142 | 0.52 | *trnH*-*GUG* |
| Leu | CUA | 301 | 0.75 | *trnL*-*UAG* | Gln | CAA | 618 | 1.52 | *trnQ*-*UUG* |
| Leu | CUG | 160 | 0.4 |  | Gln | CAG | 195 | 0.48 |  |
| Ile | AUU | 984 | 1.51 |  | Asn | AAU | 831 | 1.53 |  |
| Ile | AUC | 359 | 0.55 | *trnI*-*GAU* | Asn | AAC | 257 | 0.47 | *trnN*-*GUU* |
| Ile | AUA | 612 | 0.94 | *trnI*-*CAU* | Lys | AAA | 917 | 1.54 | *trnK*-*UUU* |
| Met | AUG | 528 | 1 | *trn(f)M*-*CAU* | Lys | AAG | 272 | 0.46 |  |
| Val | GUU | 472 | 1.47 |  | Asp | GAU | 720 | 1.61 |  |
| Val | GUC | 140 | 0.44 | *trnV*-*GAC* | Asp | GAC | 177 | 0.39 | *trnD*-*GUC* |
| Val | GUA | 493 | 1.54 | *trnV*-*UAC* | Glu | GAA | 904 | 1.51 | *trnE*-*UUC* |
| Val | GUG | 176 | 0.55 |  | Glu | GAG | 296 | 0.49 |  |
| Ser | UCU | 474 | 1.7 |  | Cys | UGU | 200 | 1.59 |  |
| Ser | UCC | 252 | 0.9 | *trnS*-*GGA* | Cys | UGC | 52 | 0.41 | *trnC*-*GCA* |
| Ser | UCA | 316 | 1.13 | *trnS*-*UGA* | Stop | UGA | 0 | 0 |  |
| Ser | UCG | 166 | 0.6 |  | Trp | UGG | 394 | 1 | *trnW*-*CCA* |
| Pro | CCU | 354 | 1.48 |  | Arg | CGU | 300 | 1.33 | *trnR*-*ACG* |
| Pro | CCC | 197 | 0.82 |  | Arg | CGC | 99 | 0.44 |  |
| Pro | CCA | 259 | 1.08 | *trnP*-*UGG* | Arg | CGA | 313 | 1.39 |  |
| Pro | CCG | 147 | 0.61 |  | Arg | CGG | 100 | 0.44 |  |
| Thr | ACU | 470 | 1.59 |  | Arg | AGA | 347 | 1.25 | *trnR*-*UCU* |
| Thr | ACC | 230 | 0.78 | *trnT*-*GGU* | Arg | AGG | 117 | 0.42 |  |
| Thr | ACA | 347 | 1.17 | *trnT*-*UGU* | Ser | AGU | 390 | 1.73 |  |
| Thr | ACG | 135 | 0.46 |  | Ser | AGC | 149 | 0.66 | *trnS*-*GCU* |
| Ala | GCU | 583 | 1.84 |  | Gly | GGU | 529 | 1.33 |  |
| Ala | GCC | 195 | 0.62 |  | Gly | GGC | 180 | 0.45 | *trnG*-*GCC* |
| Ala | GCA | 333 | 1.05 | *trnA*-*UGC* | Gly | GGA | 580 | 1.46 | *trnG*-*UCC* |
| Ala | GCG | 156 | 0.49 |  | Gly | GGG | 301 | 0.76 |  |

**Note.**

RSCU: Relative Synonymous Codon Usage.
