# Supplementary material for: The complete chloroplast genome sequence of strawberry (Fragaria × ananassa Duch.) and comparison with related species of Rosaceae
Source: PeerJ. 2017 Oct 12;5:e3919. doi: 10.7717/peerj.3919 (PMC5641433; doi:10.7717/peerj.3919)
Supplement: File S4 [file peerj-05-3919-s004.docx]

| **No.** | **Length (bp)** | **Type** | **Repeat 1 location** | **Repeat 2 location** |
| --- | --- | --- | --- | --- |
| 1 | 67 | P | IGS (*trnM*-*CAU*-*atpE*) | IGS (*trnM*-*CAU*-*atpE*) |
| 2 | 52 | P | IGS (*trnR*-*UCU*-*atpA*) | IGS (*trnR*-*UCU*-*atpA*) |
| 3 | 48 | P | IGS (*trnE*-*UUC*-*trnT*-*GGU*) | IGS (*trnE*-*UUC*-*trnT*-*GGU*) |
| 4 | 40 | F | IGS (*rps12*-*trnV*-*GAC*) | *ndhA* |
| 5 | 40 | P | *ndhA* | IGS (*trnV*-*GAC*-*rps12*) |
| 6 | 39 | F | intron *ycf3* | IGS (*rps12-trnV-GAC*) |
| 7 | 39 | P | intron *ycf3* | IGS (*trnV-GAC-rps12*) |
| 8 | 38 | F | intron *ycf3* | *ndhA* |
| 9 | 44 | R | IGS (*rps16*-*trnQ*-*UUG*) | IGS (*rps16*-*trnQ*-*UUG*) |
| 10 | 38 | P | IGS (*psbT*-*psbN*) | IGS (*psbT*-*psbN*) |
| 11 | 31 | R | IGS (*trnT*-*UGU*-*trnL*-*UAA*) | IGS (*trnT*-*UGU*-*trnL*-*UAA*) |
| 12 | 30 | P | IGS (*psbI-trnS-GCU*) | *trnS-GGA* |
| 13 | 36 | R | IGS (*trnP*-*UGG*-*psaJ*) | IGS (*trnP*-*UGG*-*psaJ*) |
| 14 | 31 | F | IGS (*rbcL*-*accD*) | IGS (*rbcL*-*accD*) |
| 15 | 33 | R | IGS (*rps16*-*trnQ*-*UUG*) | IGS (*rps16*-*trnQ*-*UUG*) |
| 16 | 33 | R | IGS (*trnS*-*UGA*-*psbZ*) | IGS (*trnS*-*UGA*-*psbZ*) |
| 17 | 31 | R | *psbJ* | *psbJ* |
| 18 | 32 | F | IGS (*rps16*-*trnQ*-*UUG*) | IGS (*accD*-*psaI*) |
| 19 | 32 | F | IGS (*psbI*-*trnS*-*GCU*) | IGS (*psbC*-*trnS*-*UGA*) |
| 20 | 32 | F | *ycf2* | *ycf2* |
| 21 | 32 | P | *ycf2* | *ycf2* |
| 22 | 32 | P | *ycf2* | *ycf2* |
| 23 | 31 | P | IGS (*accD*-*psaI*) | IGS (*accD*-*psaI*) |
| 24 | 31 | P | Intron *ndhB* | Intron *ndhB* |
| 25 | 31 | F | Intron *ndhB* | Intron *ndhB* |
| 26 | 30 | C | IGS (*rps16*-*trnQ*-*UUG*) | IGS (*accD*-*psaI*) |
| 27 | 30 | P | IGS (*rps16*-*trnQ*-*UUG*) | IGS (*accD*-*psaI*) |
| 28 | 30 | F | IGS (*rps16*-*trnQ*-*UUG*) | IGS (*ndhC*-*trnV*-*UAC*) |
| 29 | 30 | C | IGS (*rps16*-*trnQ*-*UUG*) | IGS (*ndhC*-*trnV*-*UAC*) |
| 30 | 30 | P | IGS (*trnS*-*GCU*-*trnG*-*GCC*) | Intron *clpP* |
| 31 | 30 | P | IGS (*trnS*-*GCU*-*trnG*-*GCC*) | IGS (*ndhC*-*trnV*-*UAC*) |
| 32 | 30 | F | *trnG*-*GCC* | *trnG*-*UCC* |
| 33 | 30 | P | IGS(*psbC*-*trnS*-*UGA*) | *trnS*-*GGA* |
| 34 | 30 | F | *psaB* | *psaA* |
| 35 | 30 | F | Intron *ycf3* | Intron *ycf3* |
| 36 | 30 | F | IGS (*rrn4.5*-*rrn5*) | IGS (*rrn4.5*-*rrn5*) |
| 37 | 30 | P | IGS (*rrn4.5*-*rrn5*) | IGS (*rrn5*-*rrn4.5*) |
| 38 | 30 | P | IGS (*rrn4.5*-*rrn5*) | IGS (*rrn5*-*rrn4.5*) |
| 39 | 30 | F | IGS (*rrn5*-*rrn4.5*) | IGS (*rrn5*-*rrn4.5*) |

**Notes.**

F=forward; R=reverse; C=complement; P=palindrome; IGS=intergenic spacer.
