# Supplementary material for: The complete chloroplast genome sequence of strawberry (Fragaria × ananassa Duch.) and comparison with related species of Rosaceae
Source: PeerJ. 2017 Oct 12;5:e3919. doi: 10.7717/peerj.3919 (PMC5641433; doi:10.7717/peerj.3919)
Supplement: File S6 — The phylogram of ‘combined regions’ was constructed based on MP analysis using all six regions (trnK-matK, trnS-trnG, atpF-atpH, trnC-petN, trnT-psbD, and trnP-psaJ) together. Numbers above and below nodes are bootstrap support values ≥50%. [file peerj-05-3919-s006.pdf]

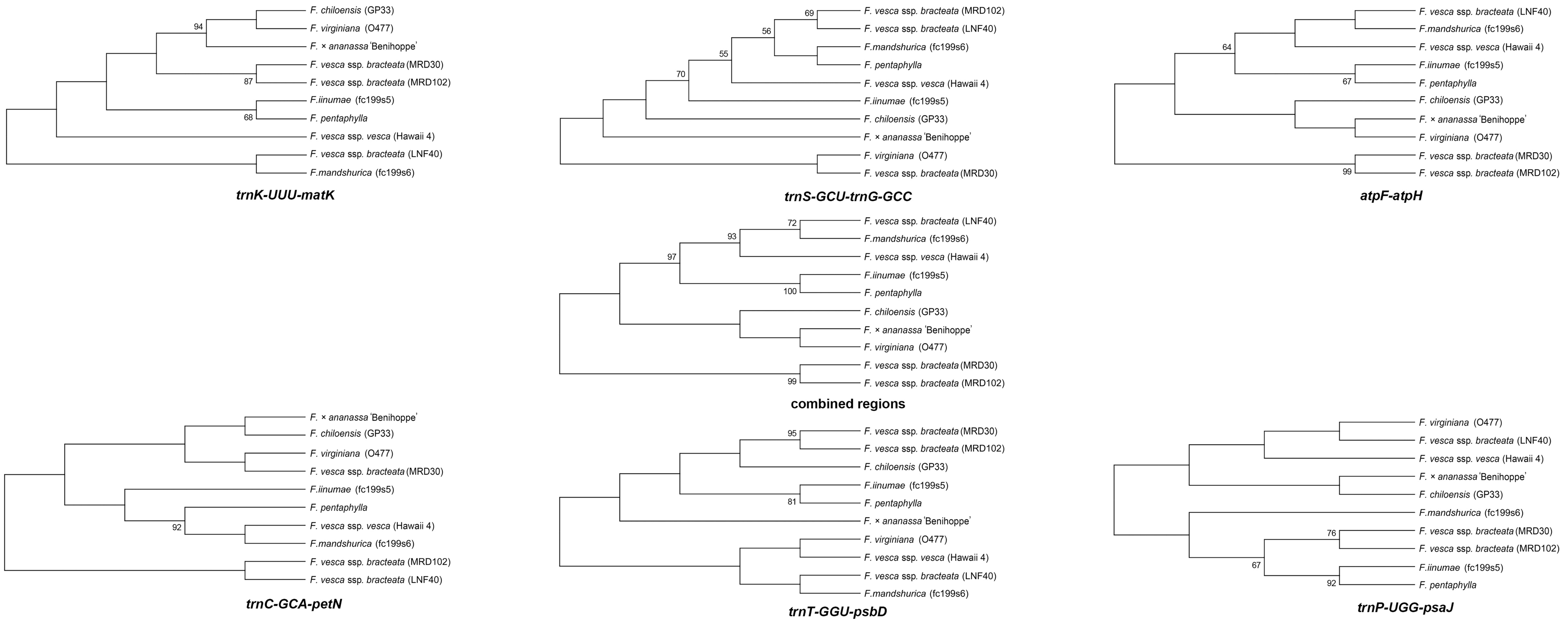

F. chiloensis (GP33)
F. virginiana (O477)
F. × ananassa 'Benihoppe'
F. vesca ssp. bracteata (MRD30)
F. vesca ssp. bracteata (MRD102)
F.iinumae (fc199s5)
F. pentaphylla
F. vesca ssp. vesca (Hawaii 4)
F. vesca ssp. bracteata (LNF40)
F.mandshurica (fc199s6)
94
87
68
trnK-UUU-matK
F. vesca ssp. bracteata (MRD102)
F. vesca ssp. bracteata (LNF40)
F.mandshurica (fc199s6)
F. pentaphylla
F. vesca ssp. vesca (Hawaii 4)
F.iinumae (fc199s5)
F. chiloensis (GP33)
F. × ananassa 'Benihoppe'
F. virginiana (O477)
F. vesca ssp. bracteata (MRD30)
69
56
55
70
trnS-GCU-trnG-GCC
F. vesca ssp. bracteata (LNF40)
F.mandshurica (fc199s6)
F. vesca ssp. vesca (Hawaii 4)
F.iinumae (fc199s5)
F. pentaphylla
F. chiloensis (GP33)
F. × ananassa 'Benihoppe'
F. virginiana (O477)
F. vesca ssp. bracteata (MRD30)
F. vesca ssp. bracteata (MRD102)
64
67
99
atpF-atpH
F. vesca ssp. bracteata (LNF40)
F.mandshurica (fc199s6)
F. vesca ssp. vesca (Hawaii 4)
F.iinumae (fc199s5)
F. pentaphylla
F. chiloensis (GP33)
F. × ananassa 'Benihoppe'
F. virginiana (O477)
F. vesca ssp. bracteata (MRD30)
F. vesca ssp. bracteata (MRD102)
72
93
97
100
99
combined regions
F. × ananassa 'Benihoppe'
F. chiloensis (GP33)
F. virginiana (O477)
F. vesca ssp. bracteata (MRD30)
F.iinumae (fc199s5)
F. pentaphylla
F. vesca ssp. vesca (Hawaii 4)
F.mandshurica (fc199s6)
F. vesca ssp. bracteata (MRD102)
F. vesca ssp. bracteata (LNF40)
92
trnC-GCA-petN
F. vesca ssp. bracteata (MRD30)
F. vesca ssp. bracteata (MRD102)
F. chiloensis (GP33)
F.iinumae (fc199s5)
F. pentaphylla
F. × ananassa 'Benihoppe'
F. virginiana (O477)
F. vesca ssp. vesca (Hawaii 4)
F. vesca ssp. bracteata (LNF40)
F.mandshurica (fc199s6)
95
81
trnT-GGU-psbD
F. virginiana (O477)
F. vesca ssp. bracteata (LNF40)
F. vesca ssp. vesca (Hawaii 4)
F. × ananassa 'Benihoppe'
F. chiloensis (GP33)
F.mandshurica (fc199s6)
F. vesca ssp. bracteata (MRD30)
F. vesca ssp. bracteata (MRD102)
F.iinumae (fc199s5)
F. pentaphylla
76
67
92
trnP-UGG-psaJ
